# Determination of acceptance criteria for geometric accuracy of magnetic resonance imaging scanners used in radiotherapy planning

**DOI:** 10.1016/j.phro.2021.01.003

**Published:** 2021-01-25

**Authors:** Henna Kavaluus, Katri Nousiainen, Sampsa Kaijaluoto, Tiina Seppälä, Kauko Saarilahti, Mikko Tenhunen

**Affiliations:** a Radiation and Nuclear Safety Authority, STUK, Laippatie 4, FI-00880 Helsinki, Finland; b HUS Cancer Center, Helsinki University Hospital and University of Helsinki, P.O. Box 180, FI-00029 Helsinki, Finland; c Department of Physics, University of Helsinki, P.O. Box 64, FI-00014 Helsinki, Finland; d HUS Medical Imaging Center, Helsinki University Hospital and University of Helsinki, B.O. Box 340, FI-00029 Helsinki, Finland

**Keywords:** Radiotherapy planning, Magnetic resonance imaging, Quality assurance, Regulatory inspections

## Abstract

**Background and Purpose:**

Magnetic resonance imaging is increasingly used in radiotherapy planning; yet, the performance of the utilized scanners is rarely regulated by any authority. The aim of this study was to determine the geometric accuracy of several magnetic resonance imaging scanners used for radiotherapy planning, and to establish acceptance criteria for such scanners.

**Materials and Methods:**

The geometric accuracy of five different scanners was measured with three sequences using a commercial large-field-of-view phantom. The distortion magnitudes were determined in spherical volumes around the scanner isocenter and in cylindrical volumes along scanner *z*-axis. The repeatability of the measurements was determined on a single scanner with two quality assurance sequences with three single-setup and seven repeated-setup measurements.

**Results:**

For all scanners and sequences except one, the mean and median distortion magnitude was <1 mm and <2 mm in spherical volumes with diameters of 400 mm and 500 mm, respectively. For all sequences maximum distortion was <2 mm in spherical volume with diameter of 300 mm. The mean standard deviation of marker-by-marker distortion magnitudes over repeated acquisitions was ≤0.6 mm with both tested sequences.

**Conclusions:**

All tested scanners were geometrically accurate for their current use in radiotherapy planning. The acceptance criteria of geometric accuracy for regulatory inspections of a supervising authority could be set according to these results.

## Highlights

• Geometric accuracy of MRI-scanners in radiotherapy planning must be evaluated.
• Phantom acquisitions with standard and clinical sequences were performed.
• Geometric distortions were determined in several volumes of interest.
• We recommend acceptance criteria for MRI-scanners in radiotherapy planning.
• Explicit and simple acceptance criteria enable effective regulatory inspections.

## 1 Introduction

Magnetic resonance imaging (MRI) has become widespread in radiation therapy planning (RTP) in the 2010s. MRI has a superior soft-tissue contrast compared to computed tomography (CT), and the contouring of planning target volumes (PTV) and organs-at-risk (OAR) is more precise in magnetic resonance (MR) images in many anatomical regions, especially in head and pelvis [1], [2], [3].

Traditionally, MR-images have been utilized in RTP through coregistration with a planning-CT. The best registration accuracy is achieved, if the patient position is the same in the MR-images as in planning-CT. Recently, the number of RTP-dedicated MRI-scanners **—** with flat tabletops and large bores for flexible patient positioning **—** has increased at the radiotherapy clinics. Such scanners have also introduced the possibility of the MRI-only RTP, where the whole RTP from contouring to dose calculation is performed using MR-images [4], [5].

MRI-only workflow in RTP has many benefits from improving accuracy of contouring to reducing the radiation dose to healthy tissues [2], [6]. In addition, the systematic error of the MRI-CT-coregistration in the traditional RTP workflow is avoided. For these reasons MRI-only RTP is used in brachytherapy for gynecological cancers and is the suggested RTP method of American Society of Brachytherapy [7]. In external beam radiation therapy (EBRT), MRI-only RTP faces two major shortcomings: the first is the lack of attenuation information for dose distribution calculation, which has been solved by using synthetic-CT-images, and the second the limited geometric accuracy.

The geometric distortions in MRI arise from both system-related and patient-induced sources [8]. The system-related distortions include B_0_-field inhomogeneities and gradient nonlinearities. The subject-related distortions comprise of magnetic susceptibility and chemical shift artefacts. Additionally, the scanning sequence parameters affect the distortions. Thus, most modern scanners allow shimming and distortion correction algorithms for distortion reduction.

In MRI-only RTP, the distortions of PTV, bony structures and body-outline directly affect the dose distribution calculation. Additionally, the distortions can violate the deformable MRI-CT-coregistration. Hence, the MRI geometric accuracy is relevant in RTP regardless of the application. According to Weygand et al. [8] the distortions should be less than 2 mm for MRI-only RTP, and Bird et al. [9] have concluded that the geometric distortions no longer prevent the usage of MRI-only in RTP of pelvis region, provided the distortions are properly addressed.

The system-related geometric distortion in MRI can be measured with a phantom of known dimensions. Many phantoms have been suggested for the purpose, most based on signal-producing markers in a signal-suppressing medium or a grid structure in a signal-producing medium [10], [11], [12]. For example, Tortef et al. [11] and Walker et al. [12] produced large-field-of-view (large-FOV) phantoms, where vitamin capsules were inserted in signal-suppressing medium. The distortions are usually determined by detecting the markers or the grid intersections in the MR-image and comparing the positions to a geometrically-accurate reference, for example a CT-image of the same phantom.

Multiple studies have also investigated the MRI geometric accuracy from RTP point-of-view as a part of MRI-only protocol commissioning or feasibility study [10], [13], [14], [15], [16]. The dose uncertainty of the whole radiotherapy process should be less than 5% [17] as inaccurate dose in PTV or OAR or both can lead to an undesired clinical response. The required level of accuracy induces the demand for quality assurance (QA) through the patient care path from medical imaging to linear accelerators. International organizations (e.g. IAEA, AAPM) have established QA guidelines and acceptance criteria for the majority of the steps in the care path. For example, the American Association of Physicists in Medicine (AAPM) report no. 83 [18] sets requirements for CT-simulators and CT-simulation (e.g. table movement and laser accuracy).

According to the American College of Radiology (ACR) accreditation protocol at diagnostic MRI, the dimensions of the head-sized ACR-phantom should not change more than 2% in a 2D plane [19]. AAPM report no. 100 [20] recommends that on MRI-scanners, which are used for treatment planning, geometric accuracy must be regarded. They also state, that the geometric distortions should be determined over a large-FOV, but do not specify the size of this large-FOV or the allowed distortion magnitude beyond the abovesaid ≤2%. Hence, the international QA guidance is lacking concerning the MRI-scanners in RTP. As the QA currently depends on the initiative of individual clinics, the inter-site comparison and regulatory inspections of supervising authorities are difficult.

This study aimed to evaluate the performance level of MRI-scanners utilized in RTP. Multiple phantom measurements were performed to define the geometric accuracy of five different MRI-scanners at separate radiotherapy clinics. Acceptance criteria for the MRI-scanners in RTP are recommended.

## 2 Materials and methods

### 2.1 Phantom

A GRADE-phantom (generic model TS1006, Spectronic Medical AB, Helsingborg, Sweden) was used for the measurements. The phantom was compatible with various MRI-scanners and contained around 1200 spherical signal-producing markers implanted into foam. The outer dimensions of the phantom were 400 mm × 490 mm × 535 mm (Width × Height × Length), and the markers defined a volume of 430 mm × 335 mm × 455 mm (W × H × L). The phantom had smaller markers in the center and a cross on the top.

### 2.2 Image acquisition

The phantom was imaged with five different MRI-scanners (hereafter denoted as A, B, C, D and E) from three major vendors. Three of the scanners had field strength of 1.5 T (A–C) and two 3 T (D and E); all the scanners had been installed between 2011 and 2018. Additionally, all the scanners had 70-cm-bores, flat tabletops, and outer set-up lasers (LAP-lasers). Details on the scanners are given in Supplementary Table S1.

On each scanner, the phantom was placed on the imaging table and the phantom’s cross was visually aligned with the scanner’s LAP-lasers. On all scanners, three sequences were acquired: a 2D fast-spin-echo (FSE), a 3D gradient-echo (GRE), and a clinical sequence. The 2D FSE and 3D GRE sequence parameters were chosen based on the GRADE-phantom manual with small alternations between scanners. The clinical sequences were pelvic imaging sequences locally utilized at each clinic. In the 2D FSE and 3D GRE sequences, the phase-encoding direction was right-left and the receiving coil the scanner’s intrinsic body-coil. A maximal FOV (300–500 mm) without moving the imaging table was used and the vendor’s 3D geometric-distortion correction was applied to each acquisition. The acquisition parameters for the used sequences are given in Supplementary Tables S2 and S3.

Repeated measurements were performed on Scanner A: the 2D FSE and 3D GRE sequences were acquired three times without moving the phantom (so-called single-setup) and seven times with new setups (repeated-setup) during a four-month period.

In addition, the phantom was imaged with CT (Siemens SOMATOM Definition AS, Siemens Healthcare GmbH, Erlangen, Germany) before and after the repeated-setup measurements. The CT-image was acquired using a FOV of 500 × 500 mm^2^ with slice thickness of 1 mm, pixel size of 0.6 × 0.6 mm^2^, image matrix of 512x512, and a peak voltage of 120 kV. The CT-image was reconstructed with a high-definition extended-FOV of 512 × 512 mm^2^ and isotropic voxel size (1 × 1 × 1 mm^3^). The CT acquisitions were visually compared to affirm the phantom stability during the measurement period.

### 2.3 Data analysis

The MRI data was analyzed with the MriPlanner-software (v1.0.46, Spectronic Medical AB, Helsingborg, Sweden). The software used a geometrically accurate reference (i.e. our CT acquisition) and deformable registration to determine the distortions in the MR-images and produced text files that contained coordinates of real marker positions and deformed marker positions. Using the marker position information and a home-written MATLAB-code (R2019a, The MathWorks Inc., Natick, MA, US), the distortion magnitudes were determined in spherical volumes around the scanner isocenter with different diameters of the spherical volume (DSV), as well as in cylindrical volumes of interest (VOI) of different lengths along the *z*-axis (i.e. along the direction of the scanner bore; see Supplementary Fig. S1). Additionally, the one standard deviation (1SD) of the distortion magnitudes were determined marker by marker between the single-setups and the repeated-setups.

## 3 Results

**Fig. 1:**
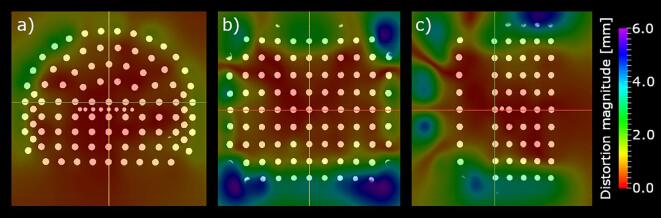
A spatial map of the geometric distortion on top of the phantom image in a) axial, b) coronal, and c) sagittal slices for the clinical acquisition of Scanner A. The slice intersections are indicated with red, yellow, and green lines. (For interpretation of the references to colour in this figure legend, the reader is referred to the web version of this article.)

An example of the measured geometric distortions as a spatial map with the corresponding phantom image is presented in Fig. 1.

**Fig. 2:**
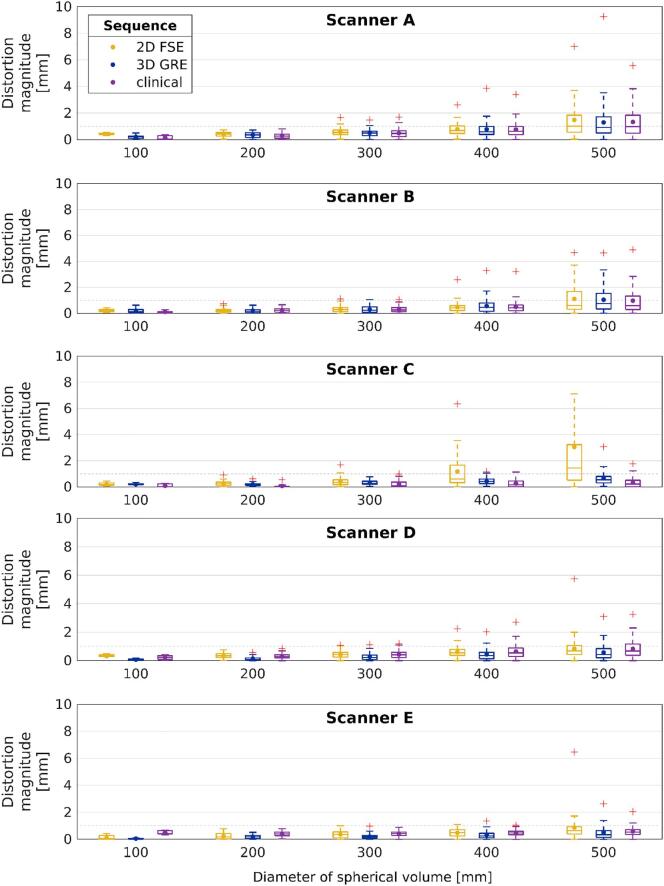
The distortion magnitude distribution at different diameters of spherical volumes around isocenter for each sequence and scanner. The dots mark the means and the lines mark the medians. The boxes define 25th and 75th quantiles, and red crosses are maximum outliers. Dashed gray line indicates distortion magnitude level of 1 mm. Scanners A-C are 1.5 T and D-E 3 T. The maximum value (16.0 mm) of 2D FSE sequence is not visible for Scanner C.

For all acquired sequences, except the 2D FSE sequence of Scanner C, the mean and median distortion magnitude was <1 mm at DSV of 400 mm, and <2 mm at DSV of 500 mm (Fig. 2). The maximum distortion was <2 mm at DSV of 300 mm for all the scanners. On Scanner C, the mean and median value of the 3D GRE and the clinical sequence was <1 mm until DSV of 500 mm, but the 2D FSE sequence had the largest mean and median distortions in DSV of 400 mm and 500 mm. Overall largest maximum distortion was 49 mm of the 2D FSE sequence on Scanner A outside DSV of 500 mm.

**Fig. 3:**
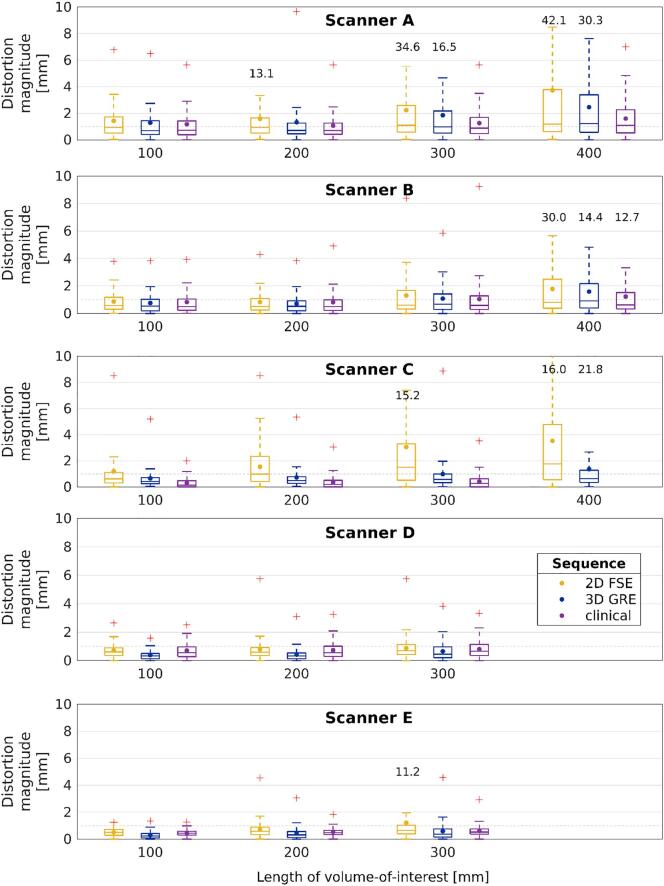
Distortion magnitude distortion versus length of volume-of-interest along z-axis. The dots mark the means and the lines mark the medians. The boxes define 25th and 75th quantiles, and red crosses are maximum outliers, of which values >10 mm are omitted for visualization purposes, but are given as text. Dashed gray line indicates distortion magnitude level of 1 mm. Scanners A-C are 1.5 T and D-E 3 T. The whisker of 2D FSE sequence ends at 11.0 mm for Scanner C.

In cylindrical VOIs of length ≤300 mm, the median distortion magnitude was <1 mm for all acquired sequences, except the 2D FSE sequences of Scanners A and C (Fig. 3). With the same sequences, the mean distortion was <2 mm in VOIs of length ≤300 mm. The maximum distortion magnitude was ≥2 mm for most of the sequences starting from the VOI of length 100 mm.

**Fig. 4:**
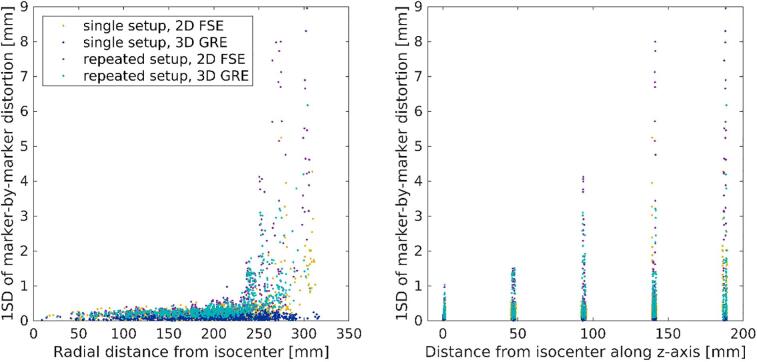
One standard deviation (1SD) of the marker-by-marker distortion magnitude for single-and repeated-setups versus marker’s radial distance (left) and distance along z-axis (right) from the scanner isocenter.

For the 2D FSE sequence, the marker-by-marker 1SD of single-setups had mean value of 0.3 mm and maximum value of 5.2 mm, and repeated-setups had mean value of 0.6 mm and maximum value of 9.0 mm (Fig. 4). For the 3D GRE sequence, the corresponding mean and maximum values were 0.1 mm and 0.3 mm for single-setups and 0.5 mm and 6.2 mm for repeated-setups, respectively.

## 4 Discussion

The aim of this study was to determine the present state of MRI-scanners in several radiotherapy clinics, and to formulate acceptance criteria for such MRI-scanners. The measurements were repeatable and the measured distortions were found to be tolerable in clinically meaningful VOIs.

For all measured scanners and sequences, except a single sequence, the mean and median geometric distortion magnitude was <1 mm for DSV of 400 mm, <2 mm for DSV of 500 mm. In cylindrical VOIs along the *z*-axis, the maximum distortion magnitudes were >2 mm even in the smallest considered VOI with length of 100 mm. The characteristic values of distortion distributions were similar for all scanners and sequences at DSV of ≤300 mm. At the DSV of 400 mm the maximum distortion magnitudes are <2% of the VOI dimensions for all scanners and sequences, which satisfy the ACR and AAPM demands. With larger distances from the isocenter differences between scanners and sequences are apparent and the distortions can increase above the 2%-threshold. The 3D GRE sequences are often more accurate further from the isocenter than 2D FSE sequences because vendor’s 3D distortion-correction algorithms function with their full capacity in 3D-volumes. The PTVs in RTP are rarely larger than 200 mm in diameter; hence, all the scanners are geometrically accurate enough for RTP with MRI-CT-coregistration.

The maximum distortion was >2 mm in all the VOIs along *z*-axis for a majority of the sequences, which could lead to a compromised geometric accuracy of a patients’ body-outline. Kemppainen et al. [21] have reported that distortion magnitude increases rapidly along the *z*-axis off the isocenter. The same phenomenon is visible in our results for VOI lengths >200 mm with several sequences. Increasing distortion must be noted in MRI-only; only short longitudinal area nearby the isocenter can be planned accurately with MRI-only [22].

Kemppainen et al. [21] reported distortions less than 2 mm in the body-outline of pelvis area and less than 1 mm in PTVs (prostate, rectum, and gynecological) and OARs (rectum and bladder). Adjeiwaah et al. [15] measured maximum distortions of 2.17 mm in 200 mm radial distance from isocenter, and Gustafsson et al. [13] mean distortions <2 mm in 200–250 mm radial distance from isocenter in phantom studies. These studies were performed with clinical sequences for MRI-only commissioning, and the dose differences between distorted and planning-CTs were <1%, when a dose difference of <2% is generally accepted [23]. Compared to these results, our measured distortion magnitudes are similar especially on clinical sequences. The clinical sequences of Scanners A, C and E were MRI-only protocols and their geometric accuracy is sufficient for MRI-only RTP. The distortions were higher in the 2D FSE sequences, which emphasizes that the MRI-only protocols must always be locally commissioned.

According to our CT acquisitions, the phantom remained stable during our measurement period. The MRI scans were repeatable within radial distance <250 mm and distance <100 mm in z-direction, where the marker-by-marker mean 1SDs of single-setups and the repeated-setups were less or equal compared to the measured distortions. Either due to the limited acquisition FOV or severe distortions, the analyses were limited to markers within <200 mm distance from isocenter in z-direction. Gustafsson et al. [13] have evaluated the intrinsic susceptibility of the GRADE-phantom, and deemed it insignificant (<0.5 mm in radian distances <250 mm measured with receiver bandwidth of 554 Hz/mm). Wyatt et al. [24] have evaluated the repeatability and set-up sensitivity of measurements with the GRADE-phantom and corresponding MriPlanner-sofware, and deemed the measurements repeatable but relatively sensitive to small (<1 mm or <1°) set-up errors. In addition, they reported that the analysis might detect markers incorrectly in the peripheral areas, if the distortion magnitude is close to the distance between the peripheral markers (ca. 30 mm). This could explain the high maximum 1SD of the marker-by-marker distortion in the single-setup and the repeated-setup measurements. Consequently, the maximum distortion magnitudes could be misidentified in the peripheral areas.

From the aspect of regulatory inspections performed by a supervising authority, the determination of geometric distortions at a couple of clinically meaningful DSVs would be the simplest solution for MRI-scanner QA. However, differences between scanners could complicate standardization of such inspections; for example Scanners D and E in this study had remarkable smaller maximum FOVs than the others. Additionally, the MriPlanner-software does not directly produce distortion magnitudes at DSVs, but rather reports with the mean and maximum distortion magnitudes in different intervals of the radial distance from the scanner isocenter (e.g. 100–150 mm) and the text files mentioned in Section 3.2. Thus, to obtain results with DSVs, further analysis is necessary when this phantom and software are used. The inspection along *z*-axis also requires further analysis, and is less robust for different FOVs. Nonetheless, defining the distortions in the cylindrical volumes provides useful information as MRI-only RTP requires accurate representation of the patient body-outline. On the downside, our measurements with GRADE-phantom cannot be used to evaluate the sequence- and patient-specific susceptibility effects.

Required geometric accuracy of MRI depends on how the MR-images are used in RTP. In MRI-only, the mean distortion should be ≤1 mm at DSV of 200 mm and ≤2 mm at DSV of 400 mm (Table 1). Very large patients should not be simulated with MRI-only due to the possibility of deformed body-outline. In MRI-CT-coregistration, maximum distortion of 2 mm at DSV of 200 mm is acceptable, which is comparable to the ACR and AAPM requirements. The anatomic region where the PTV resides must be considered; MRI is best suited for targets lying in the center of the body, whereas peripheral targets (such as breasts) suffer from distortions that could hinder the treatment accuracy [16]. Furthermore, in stereotactical radiosurgery all error sources should be less than 1% [25]. This requires excellent geometric accuracy: 1 mm in less than 2 cm PTVs and 1.5 mm in larger PTVs [26]. As the system-related distortions are 3D by their nature, the phantom measurements should be performed over a 3D volume [27], [28]. According to Ranta et al. [29] the QA measurements in 2D are sufficient once a standard level of scanner performance is established in 3D. Ranta et al. also concluded that the geometric accuracy could remain stable for years.

**Table 1:** Acceptance criteria recommendations of the geometric distortion for MRI-scanners used in radiotherapy planning: external radiotherapy to conventional target volumes (MRI-CT-coregistration and MRI-only) versus diameter of spherical volumes (DSV); external stereotactical radiosurgery to small targets according to the size of the planning target volume (PTV); and internal radiotherapy (i.e. brachytherapy).

Treatment method	Inspection volume	Distortion tolerance
MRI-CT-coregistration	DSV of 200 mm	Max 2 mm
MRI-only	DSV of 200 mm	Mean 1 mm
	DVS of 400 mm	Mean 2 mm
Stereotactical radiosurgery	PTV of <2 cm in diameter	Max 1 mm [26]
	PTV of ≥2 cm in diameter	Max 1.5 mm [26]
Brachytherapy	Region of applicator, target volume and organs-at-risk	Max 1 mm [30]

Furthermore, the geometrical accuracy is not the only feature required from an MRI-scanner in RTP. The scanner should also have a radiologically acceptable image quality and the same patient positioning precision as treatment units [27], [31]. Other properties of an MRI-scanner suitable for RTP include flat imaging tabletop, patient positioning laser system (LAP-lasers), a large bore of at least 70 cm, and large-FOV (preferably at least 50 cm). A comprehensive QA of image quality, geometrical accuracy, and mechanical properties of the scanner must be performed with regular intervals, at least once a year. The GRADE-phantom together with ACR-phantom have been found feasible for these QA measurements [32] which remain the responsibility of a radiotherapy clinic. However, even the best system-related geometric accuracy and QA measurements do not replace critical revision of the clinical images.

In the future, a regulatory inspection of MRI-scanners could consist of phantom acquisitions with standardized QA sequences as well as clinical sequences. In MRI-CT-coregistration the geometric accuracy of diagnostic-level is sufficient. In MRI-only RTP mean distortion of ≤1 mm at DSV of 200 mm and ≤2 mm at DSV of 400 mm are accepted. The presented measurements with explicit and simple acceptance criteria enable effective regulatory inspections of the MRI-scanners and the comparison of the scanners for a supervising authority. Corresponding criteria will be necessary for MR-linear accelerators and MR-image-guided radiotherapy in the future.

## Declaration of Competing Interest

The authors declare that they have no known competing financial interests or personal relationships that could have appeared to influence the work reported in this paper.
