# Supplementary material for: Determination of acceptance criteria for geometric accuracy of magnetic resonance imaging scanners used in radiotherapy planning
Source: Phys Imaging Radiat Oncol. 2021 Jan 25;17:58–64. doi: 10.1016/j.phro.2021.01.003 (PMC8058029; doi:10.1016/j.phro.2021.01.003)
Supplement: Supplementary data 1 [file mmc1.pdf]

Supplementary Material

Table S1
Information about the magnetic resonance imaging scanners used in the study.

Scanner	Model	Field strength [Tesla]	Installation year	MRI-only protocol
A	GE Optima MR450w GEM$_1$	1.5	2011	yes
B	GE Signa Artist$_1$	1.5	2018	no
C	Philips Ingenia$_2$	1.5	2016	yes
D	Siemens Magnetom Vida$_3$	3	2017	no
E	Siemens Magnetom Vida$_3$	3	2018	yes

$_1$GE Healtcare, Chigago, IL, US

$_2$Koninklijke Philips N.V., Amsterdam, Netherlands

$_3$Siemens Healthcare GmbH, Erlangen, Germany

Table S2
The acquisition parameters of the 2D FSE and the 3D GRE sequences used in the study.

Sequence	2D FSE	3D GRE
Field of view [mm^3]	500×500×320—500	500×500×352—500
Acquisition matrix	500×500 or 512×512	500×500 or 512×512
Slice thickness [mm]	2.5	1.0—2.0
Number of slices	128—200	176—508
Time of repetition [ms]	15 000	4.2—6.6
Time of echo [ms]	93—97	1.5—2.8
Echo train length	11—15	1
Flip angle [^{o}]	130	20
Water-fat-shift [pixels]	0.9—1.8	0.6—1.1

Table S3
The acquisition parameters of the clinical sequences for each scanner of the study.

Scanner	A	B	C	D	E
Sequence type	$Dixon_1$	FSE	$Dixon_1$	$Dixon_1$	$Dixon_1$
Field of view [mm^3]	500×500×500	500×500×484	546×546×300	500×520×384	500×500×352
Acquisition matrix	428×448	512×512	324×321	416×432	512×410
Phase encoding direction	right-left	anterior-posterior	right-left	anterior-posterior	anterior-posterior
Number of slices	232	484	120	320	176
Slice thickness [mm]	2.4	2	2.5	1.2	2
Slice cap [mm]	0	-1	0	0	0
Time of repetition [ms]	6.9	2502	5.7	4.3	5.6
Time of echo [ms]	2.1, 4.3	76.9	1.6, 3.8	1.3, 2.6	2.5, 3.7
Echo train length	4	88	4	4	4
Flip angle [°]	15	90	10	10	10
Water-fat-shift [pixels]	0.6	1.3	0.4	0.4	0.7
Number of averages	2	2	1	2	0.8

$_1$DIXON is a post-processing method that reconstructs multiple image sets (e.g. water and fat images) [1].

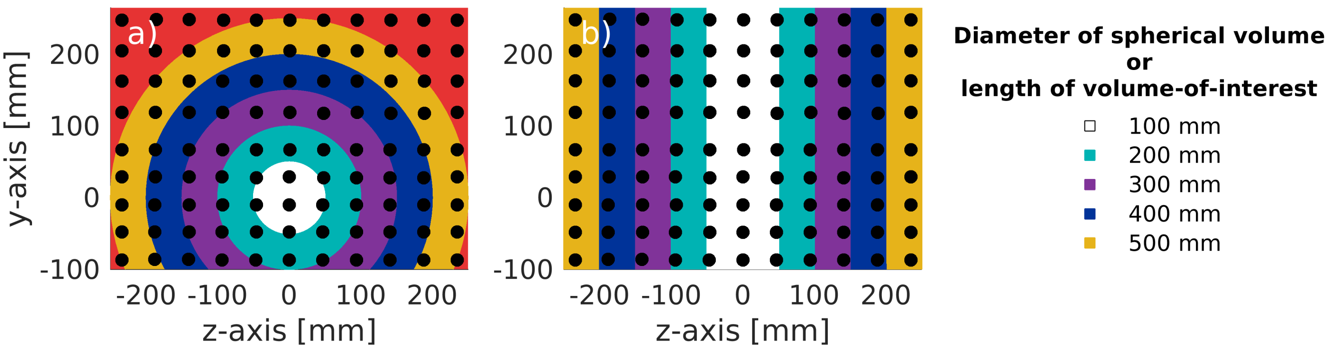

Figure S1. The approximate distribution of the signal markers (black dots) in the phantom. The color blocks represent the volumes, in which the distortion distributions were determined versus a) the diameter of spherical volume, and b) the length of cylindrical volume-of-interest along the z-axis of the scanner.

References
[1] Dixon W.T. Simple proton spectroscopic imaging. Radiology 1984;153(1):189–194.
